# Impact of MPH programs: contributing to health system strengthening in low- and middle-income countries?

**DOI:** 10.1186/s12960-016-0150-7

**Published:** 2016-08-22

**Authors:** Prisca A. C. Zwanikken, Lucy Alexander, Albert Scherpbier

**Affiliations:** 1 Royal Tropical Institute, Mauritskade 63, Amsterdam, The Netherlands; 2 School of Public Health, University of the Western Cape, Cape Town, South Africa; 3 Faculty of Health, Medicine and Life Sciences, Maastricht University, Maastricht, The Netherlands

**Keywords:** Impact, Master of public health, Evaluation, Graduate, Low- and middle-income countries

## Abstract

**Background:**

The “health workforce” crisis has led to an increased interest in health professional education, including MPH programs. Recently, it was questioned whether training of mid- to higher level cadres in public health prepared graduates with competencies to strengthen health systems in low- and middle-income countries. Measuring educational impact has been notoriously difficult; therefore, innovative methods for measuring the outcome and impact of MPH programs were sought. Impact was conceptualized as “impact on workplace” and “impact on society,” which entailed studying how these competencies were enacted and to what effect within the context of the graduates’ workplaces, as well as on societal health.

**Methods:**

This is part of a larger six-country mixed method study; in this paper, the focus is on the qualitative findings of two English language programs, one a distance MPH program offered from South Africa, the other a residential program in the Netherlands. Both offer MPH training to students from a diversity of countries. In-depth interviews were conducted with 10 graduates (per program), working in low- and middle-income health systems, their peers, and their supervisors.

**Results:**

Impact on the workplace was reported as considerable by graduates and peers as well as supervisors and included changes in management and leadership: promotion to a leadership position as well as expanded or revitalized management roles were reported by many participants. The development of leadership capacity was highly valued amongst many graduates, and this capacity was cited by a number of supervisors and peers. Wider impact in the workplace took the form of introducing workplace innovations such as setting up an AIDS and addiction research center and research involvement; teaching and training, advocacy, and community engagement were other ways in which graduates’ influence reached a wider target grouping. Beyond the workplace, an intersectoral approach, national reach through policy advisory roles to Ministries of Health, policy development, and capacity building, was reported. Work conditions and context influenced conduciveness for innovation and the extent to which graduates were able to have effect.

Self-selection of graduates and their role in selecting peers and supervisors may have resulted in some bias, some graduates could not be traced, and social acceptability bias may have influenced findings.

**Conclusions:**

There was considerable impact at many levels; graduates were perceived to be able to contribute significantly to their workplaces and often had influence at the national level. Much of the impact described was in line with public health educational aims. The qualitative method study revealed more in-depth understanding of graduates’ impact as well as their career pathways.

**Electronic supplementary material:**

The online version of this article (doi:10.1186/s12960-016-0150-7) contains supplementary material, which is available to authorized users.

## Background

The “health workforce” crisis has highlighted the need for more health (care) professionals and led to an increased interest in health professional education, including master of public health (MPH) programs [2, 14]. Recently, it was questioned whether graduates working as mid- to higher level cadres in public health were appropriately prepared to strengthen health systems in low- and middle-income countries (LMIC) [3, 9, 11]. WHO [15], in its report on transformative education, identified knowledge gaps and recommended studies on *impact* of educational programs, as well as studies on health education programs other than medical and nursing. A systematic review of evaluations of master programs in health and health care education concluded that graduate surveys were the dominant method of measurement and focused mainly on outcome [16]. Impact of training is under-reported, possibly because impact of education in general is difficult to measure, since various factors have influence: prior education might vary, workplace factors as well as the external context may cause differential potential to impact, and post-MPH education may further enhance the graduates’ competencies at the time of the study. Complex outcomes and impacts are also challenging to measure [6]. Therefore, a study combining quantitative and qualitative methods was collaboratively designed by six institutions, to measure outcome and impact, studying six MPH programs geared towards LMIC (see Table 1).

**Table 1:** Six MPH programs involved in the mixed method study

1. Royal Tropical Institute, Amsterdam, The Netherlands	
2. School of Public Health, University of the Western Cape, Cape Town, South Africa	
3. Hanoi School of Public Health, Hanoi, Vietnam	
4. School of Public Health, Fudan University, Shanghai, China	
5. National Institute of Public Health, Cuernavaca, Mexico	
6. University of Medical Sciences and Technology, Khartoum, Sudan	

Impact is divided into two components, namely workplace and sector impact, and defined as follows: changes effected at the workplace and changes effected in the sector, such as improved quality of care [16]. The quantitative results revealed that graduates were able to impact on their workplace between 20 and 60 % for 26 specific impact variables and to impact on society between 17 and 39 % for 10 defined variables [17, 18]. The qualitative component of the study was developed to study the perceived impact in more depth, in the context of LMIC. While the quantitative study includes results from all six schools, the qualitative study includes only the first two schools, where data was captured in English.

The research question therefore was as follows: what is the impact of the MPH program as perceived by graduates (2004–2010) and their peers and supervisors?

## Methods

### Study site and sample selection

The study site was two institutions: The Royal Tropical Institute (KIT), Amsterdam, where a residential MPH program located in the Netherlands is geared towards students from LMIC, and the School of Public Health (SOPH), University of the Western Cape, South Africa, a distance MPH program which is delivered from South Africa to students from over 15 African countries, many of whom choose to attend contact sessions twice annually in Cape Town.

#### Background of both schools

Students from both schools come from different countries and a wide variety of background professional education: they include medical doctors, nurses, other allied health professionals, and social scientists; all have three or more years of work experience.

The MPH program at KIT is a 1-year full-time residential program, while the program at SOPH is taken at a distance over 2 to 3 years. The cost of training was in 2006, the midpoint of the period under investigation: University of the Western Cape, ZAR 9250; KIT, 13.300 EURO (Table 2).

**Table 2:** Goals and learning objectives of KIT, Amsterdam, and SOPH, UWC, Cape Town

Institution	KIT, Amsterdam	SOPH, Cape Town
Goal	KIT’s program is designed to develop the capacity of senior health managers to use an integrated, multidisciplinary approach to addressing the health problems in their country.	SOPH’s program aims to shift mid-level health professionals from a curative/clinical orientation to a public health preventive and promotive paradigm at population level.
Learning objectives	• Analyze the health status of a community, the performance of its health care system, and the contextual factors that influence both;	• Identify, quantify, and prioritize the health problems and needs of communities;
	• Identify points at which interventions can be made to improve health and the health care system;	• Use the primary health care approach to design, implement, and evaluate comprehensive and participatory programs to address these needs;
	• Plan such interventions and implement them;	• Conduct health system research to improve quality of care;
	• Evaluate the effectiveness of the proposed activities.	• Demonstrate leadership in transforming the health and welfare systems.
Modules	2004–2009	Core modules (2004–2009)
	Determinants of Health/Introduction to Public Health	Health Development and Primary Health Care II
	Epidemiology	Measuring Health and Disease II—Intermediate Epidemiology
	Statistics	Understanding Public Health
	Organisational Behaviour and Change^a^	Health Systems Research II
	Health, Policy and Management	Mini-thesis of 7 500–20 000 words
	Health Policy and Management Planning	
	Health Promotion^a^	Plus two electives from the list below, i.e., six modules in total
	Human Resources Development	
	Health Systems Research	Elective modules (2004–2009)
	Health Policy and Financing	Stream 1—Health Promotion
	Nutrition^a^	Health Promotion II; Alcohol Problems: A Health Promotion Approach; Health Promoting Schools: Putting Vision into Practice; Health Promoting Settings: A Partnership Approach to Health Promotion
	Sexual and Reproductive Health incl. HIV/AIDS	Stream 2—Health Research
	Disease Control	Monitoring and Evaluation in Health and Development Programs; Survey Methods: Designing Questionnaires; Qualitative Research Methods; Quantitative Research Methods; Using Information for Effective Management I
	From 2007–2009: choice of last 3 modules between:	
	General	Stream 3—Health Information:
	Health Policy and Financing	Using Information for Effective Management I; Survey Methods: Designing Questionnaires; Qualitative Research Methods
	Sexual and Reproductive Health incl. HIV/AIDS	
	Disease Control	
	HIV Specialisation	Stream 4—Health Management
	Local Responses to HIV/AIDS^b^	Health Management II; Using Information for Effective Management I; Managing Human Resources for Health
	Mainstreaming HIV/AIDS^b^	
	Policy Politics and Governance of HIV/AIDS^b^	
		Stream 5—Nutrition
		Public Health Nutrition: Policy and Programming; Micronutrient Malnutrition; Epidemiology of Non-Communicable Diseases
		Stream 6—Human Resources
		Introduction to Human Resources Development in the Health Sector; Managing Human Resources for Health
		Other Electives
		Advanced Epidemiology: Measuring Health and Disease III; Children, Health & Wellbeing: A Cultural Perspective; Community Involvement in Health; Culture, Health and Illness: An Introduction to Medical Anthropology; Diet and Diseases Epidemiology and Control of HIV/AIDS & TB; Health and Social Change; Maternal and Child Health; Promoting Rational Drug Use in the Community; Women’s Health and Well-being

^a^Merged with other modules in 2006–2007

^b^Names changed

Respondent selection attempted to follow the sampling principle of maximum variation, based on key characteristics of the population (year of graduation/sex/continent); the sample was chosen from countries with the largest number of graduates for that cohort. In total, 10 graduates from KIT were interviewed and 7 from SOPH, Cape Town.

#### Sample selection, KIT, Amsterdam

Graduates were divided into two groups by graduation year: 2004–2007 and 2008–2010, by sex (M/F) and into three regions (Africa, Asia, Europe/Middle East). A graduate was randomly chosen per group from the country with the largest number of graduates for that cohort, sex, and continent. Out of the 12 KIT graduates selected, 7 had to be replaced (*) as contact attempts yielded no reply or the email address was incorrect. For two graduates, the replacement could also not be contacted (Table 3).

**Table 3:** Sample selection, KIT, Amsterdam

Continent	Africa		Asia		Europe	
Graduation year/sex	M	F	M	F	M	F
2004–2007	1*Ethiopia 2004	-*	1 Indonesia 2007	1* China 2004	1*Netherlands 2004	1*Netherlands 2004
2008–2010	1 Ghana 2008	1 Liberia 2010	1 Afghanistan 2009	1 Indonesia 2009	-*	1* Georgia 2010

*Replaced

#### Sample selection, SOPH, UWC, Cape Town

For the SOPH, the sample graduates were selected according to maximum variation criteria from five countries from which the largest numbers of students graduated between 2004–2007 and 2008–2010. Graduates were often difficult to reach resulting in a random selection within the criteria parameters. Only 7/10 graduates were reached and available. A limitation of the sampling was that although 23 % of the graduates were from Namibia, none were reachable nor those from countries where smaller numbers were located. This was, in some cases, because many of those who studied from Namibia were doctors from other countries who had subsequently returned to their home countries (e.g., SOPH-Graduate5), and in others, because a different study had been conducted in 2011 and graduates were reluctant to participate again (Table 4).

**Table 4:** Sample selection, SOPH, University of Western Cape

Country/% of graduates	South Africa (33 %)		Zambia (11 %)		Uganda (6 %)		Namibia (23 %)	Other countries (27 %)
Graduation year/sex	M	F	M	F	M	F	M	F
2004–2007	1	2		1	1		Nil	Nil
2008–2010			1	1			Nil	Nil

### Material, data collection, and analysis

Interview guides were based on the conceptual framework developed for the larger study on the outcome and impact of six LMIC MPH programs, as well as the objectives of the study [16]. The data for the other four LMIC MPH programs are not included in this study because of resource limitations for translation. The interview guides were pretested with cohorts of pre-2004 MPH graduates and revised based on the pilot results. Graduates were interviewed in 2013, which was 3–8 years after graduation, on average 5.9 years.

In-depth interviews were conducted with public health professionals who graduated between 2004 and 2010; each graduate in turn selected a peer and a supervisor from their workplace, offering multiple source feedback see Additional file 1. Interviews, which usually lasted for 1 h, were conducted by the researcher or a selected research assistant who received briefing. Most of SOPH’s interviews (90 %) were conducted face-to-face, while the location of KIT’s graduates necessitated the use of telephone and internet telephony (Skype). Research assistants conversant with the local language undertook KIT’s interviews with peers and supervisors from Indonesia and Liberia; interviews were translated and all were transcribed and imported into Atlas Ti v7.

The first six interviews (three per institution) were jointly coded by the two lead researchers to ensure consistency in coding. Coding was based initially on significant concepts related to the study questions, which were then clustered into themes and further analyzed.

## Results

The findings of this study regarding the impact of the MPH have been structured as follows: key demographic information of study participants is presented; this is followed by a table of the themes under which findings have been grouped. The over-arching themes include *impact in the workplace*, followed by a description of some *contextual factors* which affected such impact; in each section, the key codes are discussed. Thereafter, the graduates’ contribution to *impact beyond the workplace* is discussed in relation to the *external context*; finally, the *mechanisms* that were found to influence impact are also described (Table 5).

**Table 5:** Shows the key demographic information of the study participants

No. of persons interviewed	Gender	Educational background	Position at time of interview
7 alumni of SOPH-UWC	8 Male, 9 Female	4 nurses	*NGO*: communications officer and health promoter (international NGO); program advisor/researcher (international NGO); policy advisor (international NGO); coordinator and health communications officer for maternal and child health and HIV palliative care; deputy director and team leader in HIV/AIDS (national NGO); executive deputy for health (national NGO)
10 alumni of KIT		6 MD/MBBS	
		3 bachelors	
		2 bachelors social science	
		1 master public administration	
		1 physiotherapist	
			*Research institute*: senior researcher, national parastatal research center; research fellow, national research institute/hospital
			*University*: lecturer in information systems department (studying for PhD); administrator national training institution
			*Provincial and local government*: senior provincial manager: AIDS prevention, care, and treatment program; municipal manager of unit addressing the needs of vulnerable groups; head of sub-division of health facilities, provincial MoH
			*National government*: leader of division of maternal and child health, MoH; head national disease control program MoH, national consultant, MoH, and INGOs

Four short graduate profiles are offered to provide insight into the graduates’ workplaces at the time of the interview (see Table 6).

**Table 6:** Four sample graduate profiles

– SOPH-Graduate5, a medical doctor, takes a leadership role as senior manager for HIV at the national level. In this capacity, he works directly with gatekeepers (including state, traditional, and religious leaders—in particular, chiefs at the time of this research), engaging them in advocating relevant messages and processes for HIV prevention in local contexts.	
– SOPH-Graduate7 is a nurse, managing Special Programs at the local government level, monitoring and advocating the inclusion of vulnerable communities in all local government department projects—including housing, roads, water, etc.	
– KIT-Graduate3, a medical doctor, has become the national manager of a disease control program in his country, dealing with donors, responsible for projects, research and budget allocation including human resources management, salaries, and manages 90 people directly.	
– KIT-Graduate7 is a nurse and the administrator of a national training institute where nurses, midwives, and other health professionals are trained; in this role, the graduate is responsible for managing the institute, staff, and students, as well as introducing new curricula.	

Table 7 represents a summary of our results focusing on impact in the workplace and in the external context. Contextual factors are also presented.

**Table 7:** Summary of results

Themes	Codes
Impact in the workplace	Management:
	Leadership
	Innovation initiated
	Teaching and training: impact through building the capacity of others
	Advocacy
	Community engagement, including social mobilization
	Research involvement
Workplace context	
Impact beyond the workplace, at the wider societal level	Influencing policy and its development
	Capacity building beyond the workplace
	Intersectoral approach
	National reach
External context	
Mechanisms that influence impact	Respect due to MPH
	Enhanced self-efficacy
	Enhanced public health knowledge and skills

### Results regarding impact in the workplace

On returning to their workplaces, many graduates introduced new actions into their work contexts, including teaching and training, curriculum revision and development, and in-service training; they also undertook mentoring, supported program implementers, and influenced policymakers; many such actions involved advocacy and social mobilization, as well as research and support to other researchers. Post-MPH change was also reported in terms of the quality of performance, extension of roles, or revitalization of a role by incorporating a public health perspective.

#### Management: “she has been taught as a manager to lead”

Improved management and leadership capacity is an expected impact of an MPH and resulted in a number of graduate promotions to leadership positions. SOPH-Graduate3 was described thus by her peer:

… she gained a whole lot of knowledge and you see that playing out in being a much more skilled manager and knowing more … (SOPH-Peer3).

New management roles were cited, including monitoring and evaluation, national policy formulation and resource generation (KIT-Graduate1), recruitment of staff, mentoring, negotiating, leading decision-making, and improved financial management (KIT-Graduate7). Her peer corroborated that her ability to manage staff, students, and finances could be attributed to the MPH and noted that she “has grown” and is now in charge of a teaching institution for which she developed policy for both staff and students.

An expanded role was also evident in SOPH-Graduate7, who was promoted to manager of Special Programs after his MPH, responsible for 10 staff, networking, and fund raising. His supervisor highlighted the value of “his systematic approach” with regard to planning (SOPH-Supervisor7).

Coordination between the national and provincial levels, or the provincial and district levels, between programs, and between different organizations was referenced in a number of interviews. KIT-Graduate4’s coordinating role was commented on by the supervisor, graduate, and peer, who noted how this impinged on her regular roles of advising on public health policy and documenting initiatives.

Respondents from both institutions commented on graduates engaging a more consultative style of management after the MPH: they referred to leading as part of a team, a style not usual in their contexts (SOPH-Peer5, SOPH-Peer7, and KIT-Supervisor7).

Also the staff are willing to work with her because of the type of leadership style she has – that she involves her undermen in making decisions. That is also helping her to continue to work smoothly at the institution (KIT-Peer7).

Furthermore, some respondents highlighted how their MPH studies made them more sensitive to co-workers’ needs and to the importance of leading from *within* a team. SOPH-Graduate3 directly attributed this ability to the MPH course, a perspective corroborated by both peer and supervisor:

… but also leading the wider teams at national level. That was very helpful. You know but doing that course helped me to be both task orientated and people orientated.

Some graduates reportedly exerted substantial agency in their positions, suggesting a relatively high level of autonomy in the workplace: one graduate was reported by KIT-Peer5 as having educated hospital staff in human resources development, established the hospital as an education center for medical students, and arranged a private sector collaboration which resulted in a high-speed internet connection; this was attributed by his peer to “knowledge and skills [gained] in the MPH.”

A significant specific change was made by a graduate who, as country manager of a national disease program, tackled human resources policy change by negotiating with the Ministry. Through this process, he facilitated realignment of employment conditions for different cadres of workers, thereby removing competitive conflict and rationalizing functions (KIT-Graduate3)*:*

Previously we had two types of employees. One type was contracted employees, which was contracted for Ministry of Public Health only for Global Fund implementation project, but now I merged the government system and Global Fund, something like vertical system was there, but now I made a unified system for all [national program] staff …

#### Leadership

Enhanced leadership was strongly acknowledged by the graduates in the following ways: graduates took the opportunity to act as leaders, accepted assigned leadership roles, were recognized as exercising leadership, and had the confidence to work from competencies developed during the program to enhance the quality of their leadership; it was also implied that leadership involved an improved capacity for vision on the part of graduates. There was also evidence that MPH graduates were *expected* to be able to lead and that they were being fast-tracked into such positions because of their MPH qualification.

Both SOPH-Supervisor3 and SOPH-Supervisor5 commented that the graduates were employed with an expectation to lead:

“He was employed with the expectation of teaching others on leadership, those without an MPH.” [This is reiterated by SOPH-Supervisor3]: “by virtue of their training [they] are quickly fast tracked into management and leadership positions” [as it is assumed they have those competencies. SOPH-Supervisor3, however, emphasized leadership as an expected outcome of the MPH.]

The supervisor of KIT-Graduate1 commented that he showed leadership when the opportunity arose, attributing this to the MPH. Interviewees for this graduate described how he worked to convince hospital staff of the importance of public health at the regional level and represented a large NGO at the national level in debates, influencing policy and teaching authorities (KIT-Graduate1, KIT-Peer1, KIT-Supervisor1).

Team leadership capacity was acknowledged by SOPH-Graduate3 who stated that she was now able to lead a team, which in her view was the result of specific modules in her MPH; her team leadership capacity was supported by her supervisor (SOPH-Supervisor3).

SOPH-Graduate5 who manages an HIV prevention intervention working with political, religious, community, and traditional leaders felt himself to be able:

“to lead a team into taking particular directions that will culminate into effectively achieving a certain set of objectives and goals for the team.” [His peer commented]: “… He is leading a team which I would say is the largest team here” (SOPH-Peer5).

KIT-Supervisor7 comments the following:

“So she [KIT-Graduate7] is in charge of all of the instructors, of whatever goes on, of all of the three hundred and plus students that she has in her institution … I mean she is in charge of the entire entity and she makes sure that it runs day to day. And it is going on without any problem.” [KIT-Peer7 attributes her improved management to the MPH]: “… because she came back with the knowledge … brought it back for us to revise our policy for both the instructors and the student body.”

Leadership was also endorsed as instrumental and essential in project implementation: KIT-Graduate5 led a large research project; his supervisor stated that, “he has a major role in success of this project,” ensuring integrity of implementation. The same graduate was modest and did not feel himself to be a good leader but identified himself as a problem solver, dealing with different stakeholders.

Enhanced leadership was not only identified in higher echelons but was acknowledged in a range of levels in the system. KIT-Peer3 cited the graduate’s increased confidence as important.

I think he’s certainly much more confident now in his … in his stand if you see what I mean. So he is much more able to argue his case to … at the committee table; he’s become a very good chairman with complicated and fractious meetings.

Leadership at the program level was appreciated by both the peer and supervisor of SOPH-Graduate7:

“He has those qualities and the skills particularly of a good leader.” [His peer notes]: “… he came up with the [strategy]… for the staff to be united, that is unity” (SOPH-Peer7).

Impact through enhanced leadership was therefore evident at multiple levels, ranging from team to overall organizational level.

#### Innovation initiated

Apart from impacting as managers and leaders, a number of graduates from the sample had initiated notable innovations in their workplaces, some with national reach, while others focused on their facility or region. Amongst the evidence gathered, several graduates influenced policy, for example, by “work[ing] around the system” to introduce AIDS and addiction research, by setting up research in different hospitals and clinics belonging to national as well as provincial government and also establishing training opportunities for nurses in addiction and AIDS patient care:

I am a government official, but sometimes if you want to work fast and efficient it’s better using the private line – NGO … or semi-private organization. So … I talked to some of my teachers and colleagues here in x and we established an addiction and AIDS research center. So it consists of several researchers from several universities and organizations and hospitals. So … I used the … the research center as a hub for… how to say, spreading my ideas regarding HIV and addiction research … (KIT-Graduate5).

Another supervisor (SOPH-Supervisor3) described a similar practice:

… in her kind of setting you need people that can move things forward and can take risks on behalf, well calculated risks, on behalf of the organization. So she was able to set up, for example gender mainstreaming, going to partners, working out opportunities of linkages and programming that could be supported by other partners ….

Finally, SOPH-Graduate6 and KIT-Graduate10, the latter reported by KIT-Peer10, both set up information systems at the workplace level to address problems that they were experiencing in their own work processes. Certain graduates seemed able to exercise a high level of self-efficacy as individuals, but their training seems to have offered them the necessary strategies and problem-solving skills to recognize opportunities and undertake innovative actions.

#### Teaching and training: impact through building the capacity of others

A substantial number (12 out of 17) of graduates were involved in training in their own or related organizations, as well as in community contexts; in both, they provided mentoring and in-service capacity building. Notably, the role of trainer was highly respected by representatives of all three cadres of interviewees, as is evident in SOPH-Peer1’s words:

… and I understand that this year actually she was appointed honorary lecturer there, which is something that is very big for someone ….

That so many graduates were positioned to pass on to colleagues what they had learnt in the MPH was to some extent an unexpected impact: it raises the importance of *relevant* MPH training to an even greater level. A graduate nurse (KIT-Graduate7) appointed as administrator of a national training institution discussed her ability to argue for and promote a revised curriculum which had by then been accepted nationally; she attributed this to her MPH noting:

My interactions with my colleagues have also changed and I have more or less become like a master trainer.

Providing in-service training was also frequently referenced in a range of different public health areas; here, credibility amongst colleagues is likely to have been critical, in order that the incumbent could play this role: KIT-Graduate8’s role in an NGO working on TB was to:

work[ed] as a medical advisor to do the technical things like provide the training of the project, policies and also the monitoring.

KIT-Peer3 reported that another graduate runs workshops, refresher training, and sessions on new guidelines for regional and provincial managers and other people. A further example is KIT-Graduate1, who trained in the workplace around the public health perspective.

At a less formal level, about half of the sampled graduates stated that they had grown into providing a mentoring role to younger colleagues, researchers, and students, and both peers and supervisors corroborated this; furthermore, some peers and supervisors mentioned this, without the graduate having mentioned it.

Furthermore, the quality of SOPH-Graduate5’s guidance and support was directly attributed to the MPH by his supervisor:

… the way he provides guidance on what should be done to the other people in the team or even if we have our meetings here, he will provide this guidance ‘maybe I think instead of doing this direction, can we move into this direction?’ … I think that’s where his MPH really helps him (SOPH-Supervisor5).

Supportive leadership orientated to building capacity, initiating and running training, problem solving as a way of building capacity, and inspiring and mentoring others were all deemed important and valued impacts.

#### Advocacy

One of the themes that emerged from the workplace analysis is that advocacy, defined by Nutbeam [7] as “… a combination of individual and social actions designed to gain political commitment, policy support, social acceptance and systems support for a particular health goal or program,” was a strong element of many graduates’ roles, both upwards to ministry officials and outwards to stakeholders and population groupings; for many, it seemed a new practice and a very important one in initiating and managing interventions. Many participants attributed these skills directly to their MPH programs.

The mid- and high-level professional roles played by graduates meant that they were often involved in advocacy to others in the health system as part of health system reform or improvement. KIT-Graduate6 was promoted to the provincial AIDS Commission in government (with responsibility for HIV/AIDS services and planning) and attributed her ability to advocate and represent the district and governor’s position to the MPH. She noted that her role involved internal lobbying to government for a budget to address HIV/AIDS needs, through the district AIDS Commission, as well as advising the planner.

A medical doctor who had changed role from surgery to national country coordinator of a particular disease control program was described by his peer as follows:

… he is much more senior figure than he was before: it is a very public position this one; he’s quite regularly in the media, doing radio and television interviews and so on and so forth (KIT-Peer3).

KIT-Graduate7 was liaising with senior staff in town councils:

Another competence is my ability for advocacy … For example, I actually head a training institution and sometimes we do need-assessment. Which means you actually have to go, to sit and to talk and to appeal to people in order to be able to get whatever we need to get, and I advocate in that like.

Another nurse and graduate, working for an AIDS-support non-governmental organization (NGO), described how the MPH program enabled her to advocate a “settings approach” for the HIV/AIDS national strategy to the Ministry of Health and stakeholders. To do so, she drew on the MPH Health Promotion course content, an approach which she felt had been both understood and taken up by stakeholders (SOPH-Graduate3).

As part of a country’s strategy to involve a wide range of leaders—politicians and religious and traditional leaders—in the national HIV approach, a graduate’s skill in communicating and advocating new plans was highly spoken of by both his peer and supervisor (SOPH-Graduate5).

… what has been very helpful is we actually not work with the Chief alone or with his headmen but we also work with the members of parliament because they also represent the same constituents, and we realised it helps, those issues help the members of parliament now to – actually, for the members of parliament we are interested in advocacy, the things they pick from their own communities that the people realise these are issues to be taken back to parliament, why?! So that parliament can understand “this is what is happening in communities, this is what communities want” (SOPH-Supervisor5).

He was also commended for his ability to pitch his approach without regard for his own status. The same graduate made the important point of engaging in advocacy with both traditional authorities (chiefs/headmen) and those in parliament, thereby assisting parliamentarians to understand the needs of targeted communities.

#### Community engagement, including social mobilization

Community involvement is referred to as a “shift in emphasis from external agencies supplying health services, to the people of a community becoming active participants in their own health care” (Dennill, King, and Swanepoel, 1999:9, as cited by Mchunu and Gwele [5]). In other words, they become partners in health care, assessing their needs, involving themselves in decision-making, planning, and evaluating the care received. In these data, community engagement is articulated as understanding different aspects of a community, interacting with them appropriately and strategically, and communicating and undertaking social mobilization at different levels.

In almost all graduate interviews, community involvement was mentioned and often echoed by either peer, supervisor, or both. However, for some graduates, community involvement simply meant *how do we convince the community* (SOPH-Graduate3, KIT-Graduate8, KIT-Graduate9). Others implied understanding community hierarchies, identifying gatekeepers, being able to communicate and plan with community, and developing community ownership (SOPH-Peer5) but also involving other groupings, e.g., members of parliament, religious leaders, and youth (SOPH-Graduate5, SOPH-Peer5, and SOPH-Supervisor5).

“So it is systematic wherein to involve the gatekeepers, their leaders; and once you’ve convinced them they do exceptional work for you on the ground” (SOPH-Graduate5). [His peer endorses his skill in doing so]: “he knows their tune of music, he’s at their level” (SOPH-Peer5).

Another graduate demonstrated strategic community engagement *when*:

women … had to deliver their babies outside, because of the taboo [to deliver in the village]. So what we did we got in touch with the chief (KIT-Graduate1).

For some, engagement was achieved by finding specific groups, e.g., trusted women representatives to communicate with women’s groups (KIT-Graduate3); in another country, the graduate’s religious affiliation enabled her to engage with women’s religious groups (KIT-Graduate6, KIT-Supervisor6). One graduate (KIT-Graduate1), involved in the oversight of hospitals in his country, spoke of the demand on him to mobilize communities and demonstrated recognition of some key elements thereof, such as community entry skills and trust building; again, he attributed his skills to training at KIT. Interestingly, staff at an institution was also seen *as a community*, and they were encouraged by a graduate to participate in order to get buy-in, as is observed by a supervisor (KIT-Supervisor7). For one graduate, this involved much more engagement with the public health management community (KIT-Supervisor3).

Some graduates mentioned prior experience in community engagement but asserted that the MPH program strengthened these skills by helping to:

identify root causes and … work structures in communities, and linking with the health system (KIT-Graduate7).

Some worked more indirectly, for instance, by transmitting community perceptions to colleagues (SOPH-Graduate2). In discussions of community involvement, linking with other players such as health centers and NGOs was very often mentioned, as was the ability to communicate at different levels of the health system. A graduate with psychiatric training is described by his supervisor as playing an important role with regard to addiction treatment, through communicating with community organizations and community leaders (KIT-Supervisor5). Another graduate (SOPH-Graduate2) explained how he stepped into the role of identifying perceptions of communities and translated them into health promotion campaigns and messages, be it for cholera or malaria; he was appreciated for this by his supervisor:

So I think he became a great asset to the office and to the country (SOPH-Supervisor2).

Social mobilization was a practice which many of the graduates seemed to take seriously, but it was the attitudinal and process-related aspects of it which were given greatest endorsement in these interviews. This suggests that the need for social mobilization in public health action is not in question but that peers and supervisors as well as the graduates themselves see the importance of training health professionals to project an appropriate attitude and to follow processes which take account of the recipient population’s status and values. Furthermore, some attention was given to exploring the problem’s underlying contributory factors in collaboration with communities, rather than simply enunciating health action instructions: this acknowledges health services recipients’ own capacity to understand their own problems.

The peer of SOPH-Graduate3 described how she led the process of mobilizing awareness of reproductive health problems in the community, noting that she had remained active even after her retirement:

… she has so many plans, but more plans in the community, to work with these people, mobilize … she’s still moving with her knowledge, but still going to the community to help (SOPH-Peer3).

Although graduates may have been conscious of the importance of community involvement before the MPH, there is evidence that the programs reinforced graduates’ attitudes and approach to it and their engagement with communities for social mobilization.

#### Research involvement

Both MPH programs included training in qualitative and quantitative research, as well as a thesis. About two thirds of the sample had some involvement in research at a range of levels: KIT-Graduate5 had secured funding from a national institute in the United States for a study for which he was principal investigator (PI), with a large group of research staff; KIT-Graduate10 was involved at the national level in a research study on preeclampsia/eclampsia and maternal mortality; the recommendations of this study were to be presented to the national ministry of health. Two graduates were engaged in PhD studies (KIT-Graduate5 and SOPH-Graduate6) and saw research as a long-term career trajectory while SOPH-Graduate1 was working at an advanced level within a national research institute and was at the time involved in a randomized control trial on gender-based violence in schools.

A large proportion of the sample seemed to consider that research should be operational and used to inform public health action, although one graduate (KIT-Graduate3) was engaged in a biomedical study on malaria prophylactics. Different types of research were reported including qualitative research based on:

“focus-groups discussion with the extension-workers and other key informants working at the health center … about the packaging, the formulation and also the registration of medicines for malaria, malnutrition and diarrhea (KIT-Graduate2)” [after which he was repeatedly asked by United Nations agencies to conduct studies as a consultant].

Large-scale drug efficacy studies were under way with KIT-Graduate3 serving as principal investigator; SOPH-Graduate4 was involved in baseline, mid-term, and final evaluations of a nutrition intervention while SOPH-Graduate7 engaged regularly in desktop research which informed work at the local government level.

Research leadership was also evident in SOPH-Graduate2, who has been the:

focal person here for [two Tobacco Control surveys]; I’m also the focal person here for the Social Determinants of Health, the (SDH). … I’m the one in charge of all this, while I get people to implement them, I’m the Supervisor ….

Several graduates were involved in publishing their work, notably SOPH-Graduate1, SOPH-Graduate4, and KIT-Graduate3, and as stated by KIT-Graduate7:

… we actually did some research work in x and I am even a co-editor of the research we did and the articles.

Others were providing a training and mentoring role in research at their workplaces (KIT-Graduate5) or a formal teaching role in institutions of higher learning (SOPH-Graduate1).

The impact of graduates’ research contribution in the workplace was positively noted by peers and supervisors but in a number of instances extended beyond the workplace.

### The influence of the workplace context

It was apparent from those we interviewed that workplace context, although not the key topic of these findings, exerted some influence on their potential to impact: some graduates experienced extremely challenging situations such as complex political conflict and resultant resource shortages, which seemed stressful but motivating. Others pointed to certain workplace facilitators of their impact.

Challenges varied widely: in the case of KIT-Graduate3, having studied away from his conflict-affected environment for a year, his peer spoke of him as being able to see the possibility of peace and prosperity, which ministry colleagues could not see (KIT-Peer3). Other graduates cited demotivating situations, such as workplaces which could not accommodate their changed capacity; this resulted in resignation and transfer of some graduates (KIT-Graduate2, KIT-Supervisor2). Lack of recognition of their higher qualifications (SOPH-Graduate5) and low remuneration were also noted as demotivating factors (SOPH-Graduate5, KIT-Peer10). Lack of acknowledgement of the graduate’s ideas or contribution, and high dependence of colleagues who were unwilling to take full responsibility for their roles, was also reported (SOPH-Graduate1, SOPH-Graduate7, and others). Managing institutional conflict was mentioned by KIT-Peer7 as a challenge, while another graduate cited an equivalent organization which has seven or eight people performing the role he performs alone (SOPH-Graduate2).

In one instance, gender discrimination had only recently started to shift, as a result of a change in political leadership; elsewhere, the age of a woman incumbent was reported to have affected her acceptance in relatively high office. Some work contexts were regarded positively in terms of gender equality: SOPH-Graduate4 noted the increased involvement of men, for example, in family planning and HIV programs.

Certain organizational factors were regarded as positive and included experience of enabling environmental factors on their return, including a “supportive environment” (SOPH-Graduate2), supportive colleagues (SOPH-Graduate4), and supervisors (SOPH-Supervisor2, SOPH-Supervisor-3, SOPH-Graduate7). On the other hand, SOPH-Supervisor3 discussed factors that might affect a graduate being able to translate what they have learnt into their workplace:

… the organizational culture matters, it does, because it’s within the organizational culture that we get people interacting and being open to new approaches or not; and the interaction between peers, is it a teamwork kind of set up, is it team learning, is it an organizational learning ….

Workplace context was therefore a key site of impact, and although challenges affected graduates’ ability to impact, there were conditions which acted as facilitators too.

The impact at the wider societal level was more difficult for all three levels of participant to identify, but some important impacts *were* described.

### Findings on impact beyond the workplace, at the wider societal level

Influence and impact beyond the workplace, at the wider societal level, was difficult to attribute to the graduate alone, as many influencing factors may have facilitated or hampered that impact. A number of codes alluded to this influence and impact, including policy-level work, national reach, representing MoH, providing evidence for policy, health system action, and an intersectoral approach. Sometimes, the graduate was modest and it was the peer and supervisor who reported more enthusiastically, relating what the graduate had achieved. Some peers and supervisors stated that they were unable to comment on impact beyond the workplace, stating that they found it difficult to disentangle the influence of the graduate and other contextual factors.

#### Influencing policy and its development

All KIT and many of the SOPH graduates mentioned that they were involved in policy development. A large number contributed to the development of national policies:

I participated in developing the national health plan (KIT-Graduate7).

Others were involved in policy interventions related to specific programs, such as malaria, health insurance, capacity development, maternal health, tuberculosis, HIV and drug abuse, and tobacco control (SOPH-Graduate2, KIT-Graduates1, 3, 4, 5, 8, 10). This was often corroborated either by the peer or supervisor or both. Some clearly attributed their involvement to the MPH:

Like I said there was skills and knowledge that I learnt during my MPH which helped me to like work with the different structures, starting from the community, the middle structures up to that higher level [SOPH-Graduate4 was discussing the advice provided to the MoH on child nutrition].

Graduates either worked for the Ministry of Health or became responsible for liaising with the national Ministry of Health on behalf of their workplace, whether an NGO, a training/research institution, WHO, or a local service delivery entity. Some graduates were asked to contribute as members of national committees, for example, on health service integration (KIT-Graduate2). Some mentioned influencing national policy, including psychosocial support for People Living with HIV (PLWHIV) (SOPH-Graduate3). Discussing sexual violence, SOPH-Peer1 noted:

She often gets invited to go and present and also to comment on the policy issues that have been developed and draft things.

Some graduates appear to have gained authority in the external arena through their masters degree:

Now I have very close contact especially with the WHO and USAID project and lots of projects which is implemented in my country. … they are meeting in the community level, at policy level, it means there is a very big change in my position, in the professional, after I have taken this master degree (KIT-Graduate3).

Similarly, SOPH-Graduate2 reported:

Whatever they are planning, and now they know my skills and what I can bring on the table they have to involve me, unlike before … It wasn’t like that before.

KIT-Graduate1 worked as national coordinator of an NGO which provides about 40 % of health services in the country; his peer commented:

… he’s more or less the resource person within the child and maternity, when it comes to maternal health, such as the policies involved at the agency level. We have the mission level, the government level and then the private sector financing. When it comes to the mission, he is the key person that we use in such programs (KIT-Peer1).

KIT-Graduate7, as administrator of a national training institution, had to deal with donors and project proposals, as well as staff and students. Some peer and supervisors from NGOs or research institutions mentioned the interaction with the department or ministry of health (SOPH-Peer1, KIT-Peer1). Some graduates, working at the Ministry, were asked by NGOs for advice or to attend their meetings (KIT-Graduate3).

A number of graduates were involved in policy development at their workplaces, for example, developing an HIV workplace policy (SOPH-Graduate7), ensuring privacy of patients, and ensuring access to preventive services for health workers, or a policy regarding traditional birth attendants or working at a European NGO in a coalition with African NGOs (KIT-Graduate4).

#### Capacity building beyond the workplace

A substantial number of graduates were involved in teaching and training outside their organizations, as well as providing advice, for example, at local universities or colleges. Many examples of teaching in higher education institutions can be cited, amongst them SOPH-Graduate1, who was appointed honorary lecturer, teaching on the MPH for a large university, as is KIT-Graduate1. KIT-Supervisor2 noted that the graduate contributed to training at five colleges, bringing learning from the MPH on advocacy into this role. Likewise, KIT-Graduate5 participated in training for five medical schools; SOPH-Graduate2 had since completed the MPH, taught advocacy and networking at the local university, and did so:

from the modules that I get from the MPH.

A potentially even greater influence was exerted through participation in institutional and country curriculum changes. SOPH-Graduate4 helped a local university to:

set up, is it like a curriculum for their public health school.

KIT-Graduate5 identified that no training on HIV existed for nurses in his country and decided to develop and conduct such training, in addition to conducting training for addiction nurses from all over the country.

A lot of nurses in x do not know how to deal with drug users. I want to know the nurses how to deal with AIDS patients to avoid stigma, to avoid discrimination, so I established and I conduct AIDS nursing.

KIT-Graduate7 in her position as higher education institute administrative head established a Diploma in Public Health at the national level and noted that:

With the improvement in the curriculum, we know that our training activities have also improved and we put in other competencies in those curricula that were not really available in others because we observed it and we noted that there were certain things that were in the curriculum that were not really meeting our present-day health needs, public health needs … (corroborated by KIT-Peer7, KIT-Supervisor7).

A number of graduates were clearly recognized as experts in their community, at work and beyond. They were asked for advice on public health issues, either in communities or through becoming an advisor or even a member of public health NGOs, committees, or coalitions, extending their impact beyond their usual work role.

#### Intersectoral approach

An intersectoral approach is taken to mean the integration of structural sectors of government or of system functionality rather than sectors in the sense of different population groupings. Such an approach involves addressing factors which influence health beyond the clinical and are often poverty related, including the interplay of poor housing conditions, lack of water and sanitation, and economic factors which prevent the advancement of communities (Schaay [12]).

SOPH-Graduate2, working for an international agency at the country level, recounted how he was exposed to an intersectoral approach in the course of his MPH and had recently implemented this approach in the context of a workshop.

… I invited all the other sectors that I feel are important in healthcare, health services delivery to highlight their role and tell them that as much as for us that are in the health sector, if they don’t play their role we cannot achieve much; So we would now have Education with us, Water and Environment, Finance, Trade … We have this now kind of committee that we set up … (SOPH-Graduate2).

Another example is of a manager working in the welfare bureau of an Asian country, who had developed a partnership with a women empowerment unit and facilitated the interaction of the health and welfare departments (KIT-Supervisor6).

A strong example of adopting an intersectoral approach was offered by a SOPH graduate working in the local government in Special Programs and implementing the Integrated Development Plan in the local government. This cross-sector department was responsible for ensuring that the needs of vulnerable populations were addressed in all sectors (SOPH-Graduate7); although it was the local government function itself which promoted this intersectoral dimension, the graduate was a strong advocate thereof and worked on communicating the importance of the approach to other sectors.

Another example of intersectoral intervention was offered by SOPH-Graduate2, in his training of journalists to formulate health messages for the public, e.g., in advocating action in response to cholera outbreaks. Furthermore, a supervisor talked of the graduate broadening malaria control beyond the Ministry of Health to include the agricultural sector (KIT-Supervisor3).

Recognition of the importance of involving other sectors in public health initiatives was an important feature of many graduates’ consciousness and actions.

#### National reach

Although some of these items concerning impact at the national level have been previously mentioned, it is of interest that a number of graduates were involved in activities and roles with national reach. For example, KIT-Graduate2 was a national consultant on a study investigating how the Global Fund supports sexual and reproductive health, as well as advising and helping in capacity building at the national level during major health reforms (KIT-Supervisor2). KIT-Graduate5 served as a member of an expert working group on HIV/AIDS in the Ministry of Health while his supervisor (KIT-Supervisor5) reported that the methadone treatment study in which the graduate was involved would be used for lobbying purposes to the MoH. Similarly, SOPH-Graduate5’s engagement in the negotiation with traditional leaders around male circumcision also had the potential to serve as a model for national distribution of the intervention.

In one instance, the graduate’s shift from medical doctor in a surgery ward to country program manager of a national program in the Ministry of Health had considerable impact on his reach as a manager; his peer noted that he is:

a more senior figure than he was before; it is a very public position this one, he’s quite regularly in the media, doing radio and television interviews and so on and so forth. He is on a number of committees that run the Ministry of Public Health … He’s also … you know, discussing with the major donors of the country program.

SOPH-Graduate2 reported that he is involved in all national Health Promotion programs, while SOPH-Graduate3 attributed her engagement at the national level to the management training she experienced in her MPH:

Yes, because Health Management really prepares you as somebody who is going to be a manager in the sector of health, different aspects of it. … it was very-very helpful because here I was leading the teams, not just here at the head office, you know I was at head office, but also leading the wider teams at national level.

Two graduates had impact (at the provincial and national levels) within the field of training: SOPH-Graduate6 advocated to provincial government to train ancillary physiotherapy workers, receiving permission and playing a significant role in running the training which finished with a 95 % completion rate; KIT-Graduate7 took part in revising the curriculum for public health training for her country, through a national planning workshop. That there was such widespread evidence of national reach suggests some significant impact through the MPH graduates.

### The influence of the external context

Not only did graduates make an impact on their national context, but their activities were also constrained or supported by factors in the external context. The influence of the external context was mentioned by 9/10 KIT graduates and two SOPH graduates.

Policy changes at the national level were mentioned as an *opportunity* for a graduate, where maternal and neonatal health became a new national focus, and according to KIT-Supervisor10:

few people only, including KIT- Graduate10, know how to [go] about new directions.

This also signaled substantial belief in the graduate’s ability.

It was evident, from a number of data sets, that *political will* was an important external mediator of impact: a graduate complained about the non-recognition of the importance of research in the public health context where they worked, noting that, despite a change of policy in the treatment for drug users, there was no money to study this change of policy (KIT-Graduate5).

The influence of politics in the working lives of graduates was mentioned, i.e., in the appointment of people, or in the words of SOPH-Supervisor3:

Sometimes at national level things would fly, sometimes not … hitting the wall.

Political influence also manifested through *policy changes in a donor* country, meaning, in one instance, a drive for an NGO towards working more with the private sector, with which the graduate was unfamiliar (KIT-Graduate4). Other graduates explained that they were obliged to change the focus of donor proposals for political and donor-driven reasons.

Societal prejudices and dynamics related to gender and age affected graduates’ impact too: where the graduate was young and female, and society was familiar with older, usually male colleagues occupying positions of authority, the graduate was under greater pressure; she was, however, recognized by her superior as having effected changes (KIT-Supervisor6). In another country, however, being a woman was regarded as an advantage, as the president had recently committed herself to empowering women (KIT-Graduate7). Approaching vulnerable groups for HIV preventive services, i.e., identifying and approaching sex workers in a Muslim country, was, however, less easy. A graduate from a high-income country (KIT-Graduate9) mentioned difficulty in finding a job, as many people have an MPH, while at the same time economic constraints were cited as a contextual factor influencing impact.

### Mechanisms that influence impact

Apart from the influence of context which is discussed below, a number of *mechanisms* emerged as playing a role in graduates’ impact in their workplaces. The concept of mechanism refers here to Pawson and Tilley’s realist evaluation approach [8], where they identify certain social, psychological, or other interpretations acting as drivers of the perceived effects of a program.

#### “An MPH gives you respect”

At the outset, it must be said that *an* MPH appeared to be highly valued amongst participants and that simply having the qualification seemed to impact on graduates’ positioning within the workplace:

… the combination to have worked and an MPH “gives you respect” (SOPH-Graduate6).

Evidence of this respect underpinned the explanation by a number of supervisors of the need for someone with an MPH for the job: they either selected the graduate because of the qualification or encouraged a member of staff to take an MPH program; this is corroborated by graduates. Supervisors advanced some reasons for requiring a staff member with an MPH: these included the ability to influence policy, capacity to develop research proposals, and the way in which it was regarded as improving performance of the individual:

how your work might be able to influence public policy, development and programming (SOPH-Supervisor1).

One supervisor explained what afforded the MPH graduate this respect:

… it’s the way you analyse issues, but more with an MPH program, … we wanted somebody who would understand, because the kind of position we wanted was somebody … who was going to understand what is happening in the communities and without a Masters, an MPH program, people don’t associate the two (SOPH-Supervisor5).

In addition, increased requirements in certain offices necessitated an MPH qualification, for example, in the country office of WHO or as head of department at a Ministry of Health. This point was also relevant to other graduates, peers, and supervisors, including SOPH-Supervisor1, KIT-Graduate2, KIT-Peer4, and many others.

Furthermore, both graduates and supervisors showed trust in the MPH training:

For me I always told people if you attend KIT, you can work under stress and you can do anything and be successful. Because they did train us (KIT-Supervisor 7).

Another supervisor notes that the MPH gives incumbents authority in high-level transactions and negotiations:

KIT-Graduate10 is the main person working with the Minister of Health and the director - she is regarded and every time asked. [This was attributed to having an international MPH] (KIT-Supervisor10).

#### Enhanced self-efficacy

In tandem with or flowing from this increased respect from colleagues, graduates seemed to have experienced enhanced confidence and self-efficacy and a greater sense of authority within their work teams or organizations.

[W]hen you have an MPH you suddenly are the person who knows it, to who people go (SOPH-Graduate3).

A graduate from KIT spoke of enhanced confidence with regard to making recommendations on addressing maternal mortality at the national level:

… and if not my knowledge in public health for example and knowledge and experience, theoretical experience in KIT, … I am not sure I would be able to give all this, to do these studies and … to work out some recommendation for the country … (KIT-Graduate10). This is corroborated by the peer (KIT-Peer10).

Another graduate linked his own professional development with social development.

So naturally I can contribute these to our discussions and then you gain confidence, you are able to network with other professionals and naturally boost your competence and … it matters. So certainly on a personal level the program has contribute to development and definitely on professional level … That means that you are creating a certain socio-economic momentum for… for development. So, public health activities I have been … have been very helpful in the challenge of … uhm … engaging communities to collaborate on activities. In this case our contribution is beyond health and it is about mobilizing social actors for change (KIT-Graduate1).

The reciprocal effect of knowledge and confidence is also expressed by SOPH-Graduate4:

… I think having confidence, because when you have confidence you are talking about this thing, you’ve got proof that this thing works and you are even sure that what you are saying really it will work, it will convince them. So that is the confidence that I’m talking about.

This confidence was also derived from another mechanism: graduates gained an advantage over workplace colleagues in that many returned with enhanced public health knowledge and skills, as well as a qualification.

#### Enhanced public health knowledge and skills

An increased knowledge of public health concepts or public health core-disciplinary knowledge was often cited by peers and supervisors alike as an indicator of the impact of the MPH. It seemed also to have accorded some graduates more respect from colleagues and, alongside this, a greater sense of authority and leadership within their work teams or organizations (KIT-Supervisor7, SOPH-Peer6, SOPH-Supervisor7).

A peer speculated on the graduate’s promotion to team leader in a non-governmental HIV/AIDS organization, emphasizing her increased knowledge from the MPH:

[What led to her promotion?] It could be her experience; it could be part of it, her attitude, her character, her zeal, coupling with so many factors besides that. And of course her knowledge she had, maybe when we discussing, that knowledge, the way she presents issues, a convincing way … She was igniting what was there, the MPH … (SOPH-Peer3).

Such changes of knowledge were also related to research skills and core disciplinary competencies in, for example, epidemiology:

… because she learned about epidemiology, she learned about statistics that she can make use of (KIT-Supervisor7).

Many respondents recognized the importance of new ways of thinking about health—such as the health promotion approach, framing health problems within the Social Determinants of Health framework (SOPH-Graduate2), or a developmental approach to health (SOPH-Graduate3). This supervisor notes the influence of introducing a health-promotive approach both at the ministry level and amongst colleagues:

… I think he integrated well and even found better ground to deliver the different activities. For instance now there is an agreed, a great understanding, the Minister(y) of health policy on the importance of the health promotion, … instead of just insisting, insisting that they invest in a clinical medicine and so on … (SOPH-Supervisor2).

A graduate also attributed winning a grant to her approach to a sustainable livelihoods issue, which she acquired through a specific module:

… because [the module] ‘Health, Development and Primary Health Care’ helps you to, show you what things ought to be and what they are not and you are able … to kind of look at people, what they need, their rights and so on; and in the gender mainstreaming we also have those elements. Yeah, so there I was that also leading the sustainable livelihoods, we were able to write a concept for our funder … in sustainable livelihoods which eventually was funded (SOPH-Graduate3).

Not only were changes in knowledge welcomed, but the graduate’s greater problem-solving skills were endorsed for targeting “resources more efficiently”:

he’s able to distill program information in a much more meaningful way … [to] re-classify the risk of malaria transmission in different districts of (country x) in order to target our resources more efficiently … (KIT-Peer3).

It was therefore of significance that impact was to some extent driven by increased knowledge of public health, the confidence that this brought, and enhanced by the respect that such new capacities engendered amongst peers and supervisors.

## Discussion

In this study, we *defined impact in* two ways: change of workplace performance and impact beyond the workplace, i.e., impact on the health sector and improved health in society [16]. Impact, however, interacts and is influenced by a range of contextual, organizational, and individual factors, which are difficult to assess and control for. Previously reported studies of the effects of masters programs in health used mostly graduate surveys and sometimes qualitative research using graduate interviews and focus group discussions, though almost all were focused on outcome and not impact [4, 16]. Other researchers mention skills applied in the workplace, i.e., outcome, but not the result of those skills, i.e., impact [4, 16]. This is therefore the first study which reports on *impact* of masters programs using a method that can be compared with multiple-source feedback.

Concerning *impact on the workplace*, the themes which yielded the richest understanding were the following: management, leadership, innovations at the workplace, capacity building of others, advocacy, community involvement, and research involvement. Leadership was reported at multiple levels, from team to the overall organizational level. As for research, some of the graduates were making impact in promoting the importance of operational and other sorts of research. Furthermore, it was apparent that some graduates took on new roles, while, in other situations, their performance in a particular role was enhanced or more pronounced. These effects on the workplace were corroborated by peer or supervisor, or both, and there was in these interactions evidence of the authority accorded an MPH (or these specific MPHs) by colleagues.

In relation to the impact graduates were making *beyond the workplace*, the themes that stood out were policy development, capacity building beyond the workplace, supporting an intersectoral approach, and engaging in activities with national reach. That so many graduates were actually involved in policy development, often at the national level or with national reach, was surprising, but provides evidence of the reach these graduates had after their MPH program. Peers and colleagues expected and appreciated support or training provided by the graduate: this very positive outcome of the MPH programs provides a route to achieving critical mass in transforming public health delivery. These findings were significant since in reviewing the variables of the quantitative study capacity building beyond the workplace and national reach were not investigated [16].

Both the quantitative study and the literature evidenced that impact of a training curriculum is difficult to define, given the complexity of the external environment, compounded by the diversity of roles played by public health professionals in the field. However, there are some studies which have captured specific areas of impact beyond the workplace: Brooker, exploring a Nursing Masters, identifies shorter patient stay and patient and carer’s improvement in knowledge as examples of impact at the societal level (Brooker as cited by Gijbels et al. [4]). Richardson identified the external impact in graduates’ development of community, hospital and clinic programs, and graduates’ successful application for development grants. He also cited the involvement of Occupational Therapy Masters graduates in the advocacy for improved client benefits and in state regulatory legislative issues [10]. Similarly, in this study, it seems legitimate to assert that there was evidence of external influence through policy and advocacy initiatives, as well as participation in capacity building at a level beyond the workplace.

In the course of this analysis, it has been notable how the intentional and tacit interaction of the graduate and their working *context* shapes the possibility of their making an impact in their workplace. The inner workplace context, the responsiveness of peers and supervisors and the response of workplace culture to an individual’s potentially disruptive presence on return, the tensions that this exerts on workplace hierarchies and relationships are a first layer of contextual reference. These can both support and drain the returning graduate’s energy to enact new behaviors. The variation in country health systems, as well as the presence or absence of humanitarian crises, situations of extreme poverty, differences in culture, society, and political shape of the country, serves as the secondary context for that health system. It is therefore remarkable that graduates were able to apply elements of their training to the extent that they did.

Peer and supervisor support, relative autonomy, and a perceived cultural environment that welcomed new ways of doing things and innovative practices were indeed often present and seemed to reassure and motivate certain graduates. In general, amongst this group of 17 graduates, there was a relatively low level of frustration regarding challenges to enacting their new authority. The qualitative nature of this study enabled the researchers to gain insight into how context interacts with new learning.

Interestingly, the data yielded some insight into *mechanisms*, which enabled graduates to exert the influence they did [13]. The effect of a student’s strengthened sense of authority within the public health field, and their peers’ and sometimes supervisors’ sense of the same, was found to be a facilitating mechanism for impact. It was apparent that graduates’ own perception of their authority had the effect of strengthening their sense of *self-efficacy*. This was also possibly related to the status that the qualification brings but also through the graduate’s sense of being able to offer different and relevant ways of looking at the public health tasks and problems across a wide range of sub-disciplines. The presence of such mechanisms seems important to these graduates making impact primarily in their workplaces and thereby possibly beyond the workplace.

Self-efficacy is, according to Bandura [1], strengthened iteratively with efficacious enactment of competency and with others’ recognition of competence. A number of graduates were able to bring something new to their workplaces, with considerable impact. This may have been enhanced by the cultural capital generally attributed to the MPH qualification but which, in these instances, can also be attributed to the graduates’ actual performance on her or his return.

Undertaking research in this arena drives home the often enunciated limitations for *attribution* when evaluating education programs: the tension between pre-existing abilities, parallel learnings, personality and context makes the complexity of this sort of analysis highly visible. Recognizing the likelihood of some level of “gratitude” or social acceptability bias, the reflections of the graduates were strengthened and often amplified by hearing about graduate impact from peers and supervisors.

The *methodological* choice of getting data from different sources was driven by an interest in triangulating impact from several perspectives—as a measure for rigor and as a means of verifying impact but also out of curiosity as to what this method of data collection would yield. Gathering data from two additional persons with distinct roles in relation to the graduate, i.e., peer and supervisor, allowed the perceptions of diversely placed colleagues to form a richer, sometimes concurrent, sometimes contradictory picture of changes in graduates’ achievements, actions, behaviors, influences, and impacts within and beyond workplaces; hopefully, this process helped to construct a more accurate picture of impact in the workplace and broader social environment, since these environments are, themselves, highly complex. Underpinning this strategy was also the concern to study the complexities of attribution, of whether the respondent was willing to attribute influence or impact to an individual and to the MPH study program. In the process, it was evident that even in the immediacy of an interview, the respondent is often aware of this dilemma, but in the nature of an in-depth interview, there is the possibility of approaching the core issue through different questions and building up layered perceptions.

The method enhanced the depth of understanding of the types of influence and impact exerted by graduates. In addition, it allowed an understanding of some of the mechanisms through which the MPH program influenced the graduate.

### Limitations

This study has some limitations. Firstly, it was the graduates who selected their peers and supervisors, which may have constituted a bias; however, given the distances between the researcher and their locations, it was very difficult for the research team to identify the peers and supervisors themselves. Some graduates could not be traced and had to be replaced. Some graduates had changed job, or the peer or supervisor had changed: the supervisor and peer were therefore not necessarily familiar with the capacities of the graduate before the MPH. Some graduates were self-employed and therefore had no peer or supervisor. Recall bias was also possible for those who had graduated up to 7 years before the study. Some supervisors and peers expressed unease when contacted, expressing reservations about using graduates’ performance for MPH program assessment. Recipients of health services as the final representatives of “societal impact” were not interviewed because it would not have been easy to identify them, and it would have been difficult for them to attribute their experiences to a specific graduate.

In some interviews, the attribution to the MPH was questioned; other reasons for impact were offered, such as personality, earlier training, or previous workplace experience and training. In many cases, impact seemed to be the confluence of multiple streams of influence, which is why one could theorize that the MPH had functioned as a doorway through which the graduate could exert influence and impact.

## Conclusions

Graduates were able to contribute to their workplace as well as beyond it, while some even had influence at the national level. There was evidence of a shift of focus from curative to more preventive and promotive action in their work (SOPH) as well as influencing health systems (KIT) at the individual level; these findings were corroborated by peers and supervisors. Much of the impact described was in line with public health educational aims. It was not, however, possible to gauge the sustainability of the impacts or changes, although the internalized nature of these changes within graduates’ practice and their appreciation by peers and supervisors suggest that the effect may be longer term.

The qualitative study revealed an in-depth understanding of impact by graduates as well as insight into possible career pathways in the context of public health in LMIC, which may offer insights for curriculum evaluation.

The multiple-source feedback tool provided valuable and multidimensional insight into the influence and impact of MPH graduates on the workplace and health systems and enabled a richer analysis and understanding of these phenomena.

Further research could be done using complexity analysis into the different factors which influence graduates’ performance, looking into the congruence of curriculum with impact, the influence of previous training, previous fields of expertise, and workplace context as well as what mechanisms can enhance impact of masters programs.

## Additional file

Additional file 1: In-depth interview guide with graduate, peer, and line manager. (DOC 65 kb)
