# Supplementary material for: Impact of MPH programs: contributing to health system strengthening in low- and middle-income countries?
Source: Hum Resour Health. 2016 Aug 22;14(1):52. doi: 10.1186/s12960-016-0150-7 (PMC4994422; doi:10.1186/s12960-016-0150-7)
Supplement: Additional file 1: — In-depth interview guide with graduate, peer, and line manager. (DOC 65 kb) [file 12960_2016_150_MOESM1_ESM.doc]

**In-depth Interview with Graduate**

9 pages

| **Suggested sequence:** The graduate should be the first interviewed as this will offer some issues which could be further explored with colleagues; then the peer, followed by the line manager.  **Criteria for selection (maximum variation):** geographical location (urban/rural, workplace type, work role, year of graduation, prior professional role, sex, ethnicity if this matters  **Objective of the interview:**  - identify influence of MPH programme on the careers of its graduates  - identify three most applied competencies in the workplace of MPH graduates  - identify influence of the graduate on the performance at the workplace, and influencing factors  - identify contribution to sector/ society by MPH graduate, and influencing factors  **Data we are hoping to derive:**  Career history and work role, attribution to MPH  Changes in role, responsibility, financial changes, promotions, personal changes  Before/after competencies (if applicable)  View of the MPH, its value in this context; what specific competencies were valued  Contribution to the workplace, and scope of influence  Hindrances or facilitators of contribution  Views on current changes in health system  How an MPH is valued in the health system  Challenges that the MPH did not prepare you for |
| --- |

**Introduction**

Consent form

**Introductory remarks**

*Thank you for agreeing to participate. We are trying to understand the outcome and impact of our Master’s program on our graduates in the workplace and the public health sector more broadly.*

**Introduce our study.** Aim of interview (e.g. understand the nature of the graduate’s job, their competencies or lack thereof for the job, and their perception of the value of an MPH in the workplace. In essence we are trying to understand the “match between” the MPH we offer and the needs of the workplace. We recognize that we cannot assume that all the competencies to be attributable to the MPH).

**How the data will be used.** That it is not an evaluation of the graduate; that no information will be communicated between participants.

**Background and orientation**

**1. Can you give me a brief career story (description of your work situation/s) before and since you graduated with the MPH?**

Probes: - Establish whether there were changes from before MPH (or during MPH in the case of SOPH-UWC);

- What was your motivation in taking the MPH?

**2. Could you explain to me your current workplace role and level?**

**The graduate and the MPH in the workplace**

**3. Can you describe any changes that have occurred since you graduated with the MPH?**

Probes: - Promotions; changes in responsibility, role, financial rewards, technical changes.

- Could any of these be attributed to having studied the MPH?

**4. Have you changed in any way because of the Masters?**

Probes: Confidence, continuous learning, self-motivation, decision-making, social service, leadership.

**5. Can you describe any (three) competencies (abilities) from the MPH which have been most helpful in your work role?**

Probes: - Examples: public health competencies, research, management, intersectoral approach, social determinants of health orientation, specific skills or knowledge.

- Please give an example of applying that competency.
- Can you be specific? e.g. services for disadvantaged groups such as TB/HIV, MCH services, research, communication, advocacy, policy, etc

**6. Can you describe your contribution and influence in the workplace since the MPH?**

Probes:- Did this change since you graduated with the MPH?

- Can you give an example where you made a change? e.g. in systems, processes, research, education/ training, service, community (prompt only those applicable).

- Do you attribute any of this to the Master’s program? Please explain why/ why not.

**7. Did anything in the workplace (either ask participant or select one) help or hinder these contributions, i.e. help or hinder implementing a Public Health approach?**

Probes: - Interviewer could choose one example given by the graduate in question 6.

- Possible issues: own competency, own confidence, other training, peer support, workplace culture, changes in policy, gender barriers, political-economic, labour market changes.

**8. Has there been anything which the MPH did not prepare you for which you have needed for your work?**

**Influence beyond the workplace**

**9. Are there currently any important changes in public health and in the health system where you work? Have you been able to contribute to them?**

Probe: - Such a change might be the introduction of a new policy or system; a new type of service unit for a specific health problem;

- If you been able to contribute to that change, can you give an example. Has this in any way been due to what you learned in the Masters program? Please explain.

…./

**10. Do you feel you have made a contribution to Public Health beyond the workplace, or exercised some influence?**

Probe: - These might be related to community outreach, advocacy, participation in policy development, intersectoral action, collaboration across facilities, health units, formulation of an intervention, setting up a partnership, developing a research proposal, supported performance improvement of colleagues

**11. Were there any factors which helped/hindered you in making such contributions?**

Probe: **-** e.g. own competency, own confidence, other training, peer support, workplace culture, changes in policy, gender barriers, political-economic, labour market changes.

**12. Would you recommend to a colleague to follow the Master’s degree of ..x..?**

Probe:- Please elaborate.

Thank you very much for your collaboration and for setting up the interviews with your colleagues!

**In-depth Interview with Peer**

| **Suggested sequence:** The graduate should be the first interviewed as this will offer some issues which could be further explored; then the peer, then the line manager.  **Criteria for selection:** worked with the graduate at least 6 months, but preferably more  **Objective of the interview:**  - identify/triangulate influence of MPH programme on the careers of its graduates  - identify/ triangulate three most applied competencies in the workplace of MPH graduates  - identify/ triangulate influence of the graduate on the performance at the workplace, and influencing factors  - identify/triangulate contribution to sector/ society by MPH graduate, and influencing factors  **Data we are hoping to derive:**  Length of time worked together; Relationship; Understanding of the role played by the graduate  Before/after MPH competencies (if applicable)  MPH and its value in this context  Graduate’s strengths and gaps in competencies  Changes in the graduate; influence in the workplace  How graduates impact on their workplace and beyond |
| --- |

**Introduction**

Consent form

**Introductory remarks**

*Thank you for agreeing to participate. We are trying to understand the outcome and impact of our Master’s program on our graduates in the workplace and the public health sector more broadly.*

**Introduce our study.** Aim of interview (e.g. understand the nature of the graduate’s job, their competencies or lack thereof for the job, and your perception of the value of an MPH in the workplace. In essence we are trying to understand the “match between” the MPH we offer and the needs of the workplace. We recognize that we cannot assume that all the competencies to be attributable to the MPH).

**How the data will be used.** That it is not an evaluation of the graduate; that no information will be communicated between participants.

**Preliminary questions to warm up**

1. How long have you worked with x? Did you work together before s/he did the MPH? What is your work role relationship?

**2. What is your knowledge of the MPH course taken by the graduate?** (Here try to establish if they have done an MPH without causing offence).

Probe: - Do you feel that it has had any influence on the way they perform their role? (Ask this in general, then gain more detail through the questions that follow).

**The graduate in the workplace**

**3. Can you talk about ..x.. since s/he graduated or since you started working with him/her?**

Probe: - If you worked with ..x.. before the Master’s and after- did you see any change, can you elaborate?

4. What do you appreciate about the way in which ..x.. works in your workplace?

Probes: Application of public health competencies, e.g. attitude, initiated changes in priorities, research, education/ training, service, community (prompt only those applicable).

**5. Has the graduate changed role or responsibilities?**

Probes: - Please explain how?

- What led to these changes?

- If there were changes, can you attribute this to ..x.. having the Masters degree? Please elaborate. Can you be more specific? - Can you give examples?

**6. Can you give an example where ..x.. made a change/ something new in the workplace since completing the MPH?**

Probes: - For example: in research, systems, processes, planning, education/ training, service, community (prompt only those applicable).

- Ask the peer to choose one workplace example (either ask participant or select one): What factors helped/hindered ..x.. in the implementation of these examples? e.g. a competency, confidence, other training, peer support, workplace culture, changes in policy, gender barriers, political-economic, labour market changes.

**7. Please can you talk about ..x.., has ..x.. changed in any way as a person since the Master’s?**

Probe: - Confidence, continuous learning, self-motivation, decision-making, social service, leadership.

**8. Are there any challenges in your workplace which you collectively could be better prepared for?**

Probe: - Can you give an example?

**Influence beyond the workplace**

**9. What are the important current changes in the Public Health system? Has the graduate been able to initiate anything in response to these changes?**

Probes: - Can you give examples? (Has this in any way been due to what ..x.. learned in the Masters program? Please explain).These might be related to community outreach, advocacy, participation in policy development, intersectoral action, collaboration across facilities, health units, formulation of an intervention, setting up a partnership, developing a research proposal, supported performance improvement of colleagues …/

- Choose one example (offered by the participant): What factors helped/hindered ..x.. in the implementation of these examples? Probe: competency, confidence, other training, peer support, workplace culture, changes in policy, gender barriers, political-economic, labour market changes.

**Concluding question**

**10. If the peer does not have an MPH, would they be interested to follow the Master’s degree of ..x..?**

Probe: **-** If yes/ no – please elaborate. If the peer has a MPH, prompt towards similarities/ differences.

Thank you very much for your collaboration!

**In-depth Interview with Line Manager**

| **Suggested sequence:** The graduate should be the first interviewed as this will offer some issues which could be further explored; then the peer, then the line manager.  **Criteria for selection:** worked with the graduate at least 6 months, but preferably more  **Objective of the interview:**  - identify/triangulate influence of MPH programme on the careers of its graduates  - identify/ triangulate three most applied competencies in the workplace of MPH graduates  - identify/ triangulate influence of the graduate on the performance at the workplace, and influencing factors  - identify/triangulate contribution to sector/ society by MPH graduate, and influencing factors  **Data we are hoping to derive:**  Length of time worked together; Relationship; Understanding of the role played by the graduate  Before/after MPH competencies (if applicable)  MPH and its value in this context  Graduate’s strengths and gaps in competencies  Changes in the graduate; influence in the workplace  Line manager’s understanding of an MPH and its value in this context  How graduates impact on their workplace and beyond  Whether line manager has studied Public Health (for context) |
| --- |

**Introduction**

Consent form

**Introductory remarks**

*Thank you for agreeing to participate. We are trying to understand the outcome and impact of our Master’s program on our graduates in the workplace and the public health sector more broadly.*

**Introduce our study.** Aim of interview (e.g. understand the nature of the graduate’s job, their competencies or lack thereof for the job, and your perception of the value of an MPH in the workplace. In essence we are trying to understand the “match between” the MPH we offer and the needs of the workplace. We recognize that we cannot assume that all the competencies to be attributable to the MPH).

**How the data will be used.** That it is not an evaluation of the graduate; that no information will be communicated between participants.

**Preliminary questions to warm up**

1. How long have you worked with x? Did you work together before s/he did the MPH? What is your work role relationship?

**2. What is your knowledge of the MPH course taken by the graduate?** (Here try to establish if they have done an MPH without causing offence).

Probe: - Do you feel that it has had any influence on the way they perform their role?

**The graduate’s role in the workplace**

**3. Can you talk a little about .. x .. in their workplace please**. *[Conversational, try to get the line manager to guide the conversation initially to see what topics are chosen]*

**4. Can you tell me a bit about her/his work role please?**

Probe: - What (MPH) competencies are most necessary in this graduate’s workplace?

- Are there any competencies that their prior professional or clinical training would not equip them for?
- Do you think s/he is challenged or under-challenged in the job? Can you explain why you say so?

**The graduate in the workplace**

**5. During the time you have worked with the graduate, have you seen any changes in the way they work?**

Probe: - Any changes in role? Changes in responsibilities, attitudes, ways of working with others? Please explain how? What might have led to these changes?

- Have there been any changes which you can attribute to ...x having studies the MPH?
- Any there any particular strengths they show, such as confidence, continuous learning, self-motivation, decision-making, social service, leadership.

6. Which (3) most important competencies or abilities does the graduate demonstrate in the roleb?

**7. Are there any critical competencies (skills, knowledge or application of them) which**

**the graduate needs, but does not have?**

Probe:

- Would you expect these to be developed during an MPH?

**8. Can you talk a little about the graduate’s influence within the workplace?**

Probe: - Has s/he taken any leadership positions relating to Public Health?

- Is there anything s/he’s done which has contributed positive change in the workplace? E.g. in research, education/ training, service, community outreach (prompt only those applicable).

- Are there any limits on the extent of influence?

**9. Are there any factors which hinder the application of a Public Health approach in this workplace?**

Probe: - e.g. missing competencies, professional hierarchies, lack of academic skills, attitudinal issues relevant to Public Health, workplace priorities and influences?

…./

**Influence beyond the job**

**10. What are the current changes in the Public Health system? Has the graduate been able to initiate anything in response to these changes?**

Probe: - Can you give examples? Has this in any way been due to what ..x.. learned in the Masters program? Please explain.

**11. Can you talk a little about the graduate’s influence beyond the workplace?**

Probe: - Is there anything s/he’s done which has contributed positive change in the community or other areas of the health system? e.g. in initiating community actions, advocacy, intersectoral linkages.

- Are there any limits on the extent of influence?

## Closing questions

12. Compared to others who do not have the MPH, do you see any differences?

Probe: - Can you elaborate please?

- Is there anything essential which should be taught in a MPH?

**13. Would you send anybody to this Master’s program? If yes/no please elaborate.**

Thank you very much for your collaboration!
